# Discovering why people believe disinformation about healthcare

**DOI:** 10.1371/journal.pone.0300497

**Published:** 2024-03-21

**Authors:** Joey F. George

**Affiliations:** Ivy College of Business, Iowa State University, Ames, IA, United States of America; SGH Warsaw School of Economics: Szkola Glowna Handlowa w Warszawie, POLAND

## Abstract

Disinformation–false information intended to cause harm or for profit–is pervasive. While disinformation exists in several domains, one area with great potential for personal harm from disinformation is healthcare. The amount of disinformation about health issues on social media has grown dramatically over the past several years, particularly in response to the COVID-19 pandemic. The study described in this paper sought to determine the characteristics of multimedia social network posts that lead them to believe and potentially act on healthcare disinformation. The study was conducted in a neuroscience laboratory in early 2022. Twenty-six study participants each viewed a series of 20 either honest or dishonest social media posts, dealing with various aspects of healthcare. They were asked to determine if the posts were true or false and then to provide the reasoning behind their choices. Participant gaze was captured through eye tracking technology and investigated through “area of interest” analysis. This approach has the potential to discover the elements of disinformation that help convince the viewer a given post is true. Participants detected the true nature of the posts they were exposed to 69% of the time. Overall, the source of the post, whether its claims seemed reasonable, and the look and feel of the post were the most important reasons they cited for determining whether it was true or false. Based on the eye tracking data collected, the factors most associated with successfully detecting disinformation were the total number of fixations on key words and the total number of revisits to source information. The findings suggest the outlines of generalizations about why people believe online disinformation, suggesting a basis for the development of mid-range theory.

## Introduction

Since the 2016 U.S. election, there has been a dramatic rise in the spread of false information, in many domains, ranging from politics to health [1, 2]. Since 2020, a significant amount of false information, or misinformation, about health has focused on COVID-19 vaccines–the quality of their development and testing, their side effects, and the motivations of governmental and corporate actors. Health misinformation has been defined as “any health-related claim of fact that is false based on current scientific consensus [3]).” Much misinformation is spread by actors who believe it to be accurate even when it is not. However, a type of misinformation–disinformation–is spread by actors who know it to be false. Disinformation has been defined as “all forms of false, inaccurate, or misleading information designed, presented and promoted to intentionally cause public harm or for profit [4].” The key part of this definition specifies that disinformation is intentional and that there is a purpose behind it. Disinformation about COVID-19 vaccines exists within a larger universe of disinformation about vaccines generally, which itself exists within a larger universe of disinformation about health and healthcare [5].

According to the Center for Countering Digital Hate [6], up to 65% of anti-vaccine content in social media originates with twelve individuals the CCDH calls the “Disinformation Dozen.” At the top of the list is Joseph Mercola, an osteopathic physician. Dr. Mercola employs dozens of people to manage his extensive social media presence, and he has a long history of disseminating healthcare disinformation. In 2012, he urged people to buy his tanning beds, arguing that the beds reduced the chances of getting cancer. He was ordered by the FTC in 2017 to refund $2.95 million (USD) to customers who had purchased the beds [7]. Dr. Mercola’s motives appear to be driven by profit, and the other members of the Disinformation Dozen appear to have similar motives. Yet acting on disinformation about healthcare can result in real physical harm. For example, a family in Florida was arrested in 2020 for selling bleach as a “Miracle Mineral Solution” for a multitude of health problems, a cure with potentially dire consequences [8]. Through August 2020, at least seven people in the U.S. died from drinking the “Miracle Mineral Solution” cure [9].

A review of the disinformation literature [10] featured a framework that crossed the dimensions of disinformation motive, facticity (the degree to which content relies on facts), and verifiability. The dimensions under the profit motive–clickbait, pseudoscience, and fake reviews–provide a multi-faceted toolkit for those, like the Disinformation Dozen, who would dupe others about healthcare for their own profit.

Disinformation about healthcare, whether clickbait, pseudoscience, or fake reviews, can result in real harm to those who act on it. Widespread disinformation can have potentially alarming consequences for individuals and adverse effects on public health [3, 11, 12]. It is important, then, to understand how people derive and evaluate disinformation in the media they consume and to understand the factors that lead them to believe and share disinformation online [13, 14]. For media posts where people believe disinformation or do not detect it, we need to understand which elements of the disinformation led them to believe and to potentially share or act on the disinformation. The study reported in this paper was designed to investigate which elements of the posts containing disinformation influence people to either believe or disbelieve them, as stated in the following research question:

RQ: What factors influence people’s evaluation of the veracity of online disinformation about healthcare?

The literature on the factors that influence the evaluation of veracity of online information dates back to studies of the veracity of websites in the 1990s [15, 16]. Studies about the veracity of information in social media sites began to appear in the literature a decade later (e.g., [17]), given that Facebook began in 2004 and Twitter in 2006. As mentioned previously, academic interest in fake news and misinformation increased in 2016 with the U.S. presidential election and continued to grow [2, 3]. Across this literature, the factors that influenced veracity assessments tended to be either individual differences in the viewers of the online information (e.g., gender, need for cognition) or characteristics of the online messages themselves (e.g., source, layout, platform). As there is no extant theory regarding how people evaluate online disinformation, the current study measured select individual differences and varied characteristics of the social media posts, as reflected in the literature. In the study, participants were exposed, one at a time, to 20 actual social media posts, some of which were true and some of which were false. They were asked to determine whether each post was honest or dishonest, true or false, real or fake, and to vocalize about their reasoning. Meanwhile, their gaze was recorded by an eye tracker in order to observe the relative importance of the characteristics of the social media posts they fixated on. The study was data-based and inductive, in that the goal was to discover aspects of human behavior rather than to test hypotheses based on extant theories.

## Literature review

### Research on health communication and disinformation

The scope of research on misinformation and disinformation in the health communication field is broad. Specific areas of research in health communication include, among other topics, recommendations for public health information specialists for communication campaigns [18]; training healthcare professionals to address misinformation their patients believe [19]; addressing misinformation about tobacco products [20]; and investigating corrective messaging on social media [21]. Related empirical work has addressed, among other topics, vaccine misinformation on Twitter [22]; the role of bots in spreading vaccine misinformation on Twitter [23]; HPV (human papillomaviruses) content on Pinterest [24]; differences in social networks in spreading misinformation and evidence-based information about Zika on Twitter in 2016 [25]; misinformation about breast cancer on Pinterest in 2018 [26]; and misinformation about CBD (cannabidiol) in GoFundMe campaigns [27]. Most of the studies cited here are data-based, extracting their findings from the data found on specific social media platforms, rather than theory-based. In addition to utilizing other research methodologies, the research on health information also employs eye tracking [28–30].

Chou and colleagues [3, 12] call for relevant research on health misinformation in five areas: 1) enhanced surveillance of the social media misinformation environment; 2) increased reliance on theory from the social sciences; 3) increased research on the effects of exposure to misinformation; 4) more research on identifying factors that may increase susceptibility to misinformation; and 5) interdisciplinary research to identify optimal strategies for responding to misinformation. The study described here addresses three aspects of their calls for relevant research: point 2 on the social sciences, point 3 on exposure, and point 4 on susceptibility to misinformation.

### Assessing credibility: Individual differences and message characteristics

Research on assessing the credibility of online content originated in the late 1990s and the early part of the following decade [15]. A large study of 2684 people’s evaluations of the credibility of websites found that the most referenced indicator of credibility was the “design look” of the website, mentioned 46.1% of the time [16]. After making empirical observations about credibility, Fogg [31] later codified his lab’s findings in Prominence Interpretation Theory, where he listed several factors as potentially influencing observers’ views on credibility. Fogg suggested that the antecedents to credibility included such factors as user involvement, website topic, user task, user experience, individual differences, user assumptions, user skills, and context, but he maintained this list was only advisory and was not to be considered comprehensive.

While Fogg and his colleagues focused on the credibility of websites in the early days of the Internet, other researchers in this time frame investigated credibility in the Internet itself, before the advent of social media. Metzger and Flanagin [32] focused on individual differences and their relationships to beliefs about Internet credibility. Looking at demographics, they found that older, female, and more educated Internet users were most concerned about credibility. People who used the Internet more frequently were less concerned and trusted online information more. There were also differences based on personality traits: Adults with a greater need for cognition–defined as “the degree to which people engage in and enjoy thinking deeply about problems or information and, thus, may be willing to exert effort to critically evaluate information ([32], p. 454)”–expressed greater concern about credibility, and users with higher faith in intuition were more likely to believe that Internet information was credible.

With the advent of social media, the focus of this stream of research shifted from assessments of Internet and website credibility to “fake news,” misinformation, disinformation, their dissemination, and the reasons people believe false content [33]. Studies of the dissemination of misinformation show that it has spread rapidly over time, especially on Twitter [34]. These findings also apply to disinformation about healthcare [5], including the prominent role played by Twitter. The spread of disinformation about healthcare has been related to increased anxiety, depression, and emotional exhaustion in social media users [35].

Why do people believe disinformation spread by social media? The research has examined a wide range of factors. Tandoc [36], basing his work on Berlo [37], cites four factors: sender (e.g., from a friend or from a stranger), message (e.g., how popular is the message, based on the numbers of likes and shares), channel (open, such as Twitter, or closed, such as WhatsApp), and receiver (e.g., selective exposure to information, which activates a positive confirmation bias). People can be persuaded to believe disinformation when it is accompanied by government censorship, due to confirmation bias, when they lack the technical skills that would allow them to better identify disinformation, and when disinformation is presented as part of ‘soft news’ (the Oprah effect) [38]. Soft news media include entertainment news shows, newsmagazines, and daytime (such as Oprah’s show, which ran on American television for 25 years) and late-night talk shows [39]. Research shows viewers are as influenced by soft news as they are by hard news, at least with respect to voting behavior [39], regardless of the content of the information. People spread disinformation on social media because they believe the stories they read and because they have some familiarity with them [40].

Empirical research on why people believe disinformation references such factors as trust in the network, beliefs about media credibility, intention to share [41], and need for cognition [42]. Appeals to emotion contained in disinformation, and one’s own emotional state during exposure, have been found to affect the extent to which people believe disinformation [43]. Appeals to emotion can distract people from analyzing information rationally. Both happy and angry moods, and feelings of social exclusion, can make people more susceptible to believing disinformation [43]. The case for the importance of the credibility and authority of the source is mixed. While some studies found source to not be important for evaluating disinformation [40, 42], other studies found the opposite. These studies found that generic sources influenced participants’ beliefs about the veracity of the disinformation they were exposed to. Websites were judged to be more credible when authored by a reliable source (content area experts vs. high school students) [44]; study participants spent more time viewing posts from high credibility sources (reputable newspapers vs. tabloids) [45]; and study participants were more anxious and felt higher crisis severity after receiving corrective information when the source was a government agency (CDC) or news media (Reuters), as opposed to an individual (Facebook friend) [46]. In general, in past research, individual differences seem to have been more important in assessing the credibility of disinformation than characteristics of the social media post [40–43].

### Disinformation and eye tracking

People process social media posts very quickly, so eye tracking, which captures 60 images per second at 60Hz, is a useful tool for observing such a rapid process. A study by Facebook found that people spent, on average, 1.7 seconds on social media content on a mobile device, vs. 2.5 seconds on a desktop [47]. In an eye tracking study, Simko and colleagues [48] found that study participants were able to browse 41 short posts in 5.1 minutes (or one every 7.5 seconds). They then followed links to seven stories, which they then read in seven minutes, or about one minute per story. Accordingly, social media consultants state that the most effective social media posts are short, and they have specific recommendations as to what that means: for example, 1 to 80 characters in a Facebook post, 25 words in a LinkedIn post, Instagram reels of 7 to 15 seconds with captions of 135 to 150 characters [49]. Given the brief amount of time which people spend with individual social media posts, some health communication scholars [28, 30] have called for viewing attention to social media posts and their evaluation through the lens of dual processing theories of communication persuasion, specifically the Elaboration Likelihood Model (ELM) [50]. ELM holds that people process communication via one of two routes, through careful and thoughtful consideration of the merits of the information presented (central), or though simple cue-based evaluation without scrutiny (peripheral). ELM is one of many dual processing models [51–54]. Although these models use different nomenclatures and posit different mechanisms, they all share in common a model with two modes of evaluation, one based on heuristics and hence fast, and the other based on systematic and controlled analysis, and hence slow. Chou and colleagues [28] found that study participants viewed target posts on cancer twice as long, on average (6.86 seconds), compared to distractor posts that were not about health (3.43 seconds). They proposed that this longer dwell time on posts may have signaled careful message processing.

Eye tracking has also been used to investigate disinformation and how people process it. In one set of studies, participants read text-only news headlines [55] or headlines first, followed by complete news stories [48, 56, 57]. Research participants were able to successfully distinguish between false and true news stories, with success rates as high as 74% [56]. However, the relationship between time spent viewing the experimental stimuli and the successful detection of false information is not clear. One study found extensive attention to a story was related to misclassifying its veracity [56]; another study found more attention to articles was related to more accurate classifications [48]. In addition, study participants had fewer fixations on false headlines compared to true headlines [57]. In other studies, participants viewed simulated Facebook news feeds [28, 29, 45] or Twitter feeds [30]. Across the studies, participants fixated longer on posts from reliable sources [45] and on posts from individuals compared to posts from organizations [28]. They fixated more on posts about cancer compared to posts about other topics [28], but their attention to the source of the post was not related to their trust in the source [29]. Kim and colleagues [30] presented participants with one post including misinformation about HPV, followed by a correction post, which was either humorous or not. They found that participants paid more attention to the misinformation text and the correction image (a cartoon) when the correction post was humorous. The non-humorous correction post was judged to be more credible than the humorous correction.

### Disinformation and cognitive processing

A related line of research has investigated the cognitive processes that people rely on to determine the veracity of social media posts. This research is also very recent, and the overall findings are not yet clear. While tagging a post as disinformation seems to have no effect on its perceived veracity, readers consider posts that have not been tagged as validated, because they haven’t been tagged [58]. Further, people who read an untrue post on social media are likely to believe it when they see it a subsequent time [59]. However, the relationship between the tendency to believe disinformation and political partisanship is not clear–one study found such a relationship [60], while another concluded that susceptibility to disinformation was driven more by lazy thinking than by political ideology [61].

### Summary

There is currently no extant theory of how people evaluate disinformation, why they choose to believe it, and what the key factors are that influence that belief. Much of the research reviewed here consisted either of literature reviews [5, 35, 38, 43] or reported on inductive research [34, 40, 42, 48, 55–57], with empirical observations based on the interaction of a set of likely variables. These observations can serve as the basis for generalizations that can eventually lead to theory [62]. The current study is also inductive, with a focus on allowing study participants to report (both through self-reports and eye tracking) those characteristics of social media posts that led them to either believe or not believe the disinformation. The factors that emerge from participant reports can then be compared to how successful they were at detecting disinformation, helping to discover generalizations about observation and belief.

**Fig 1:**
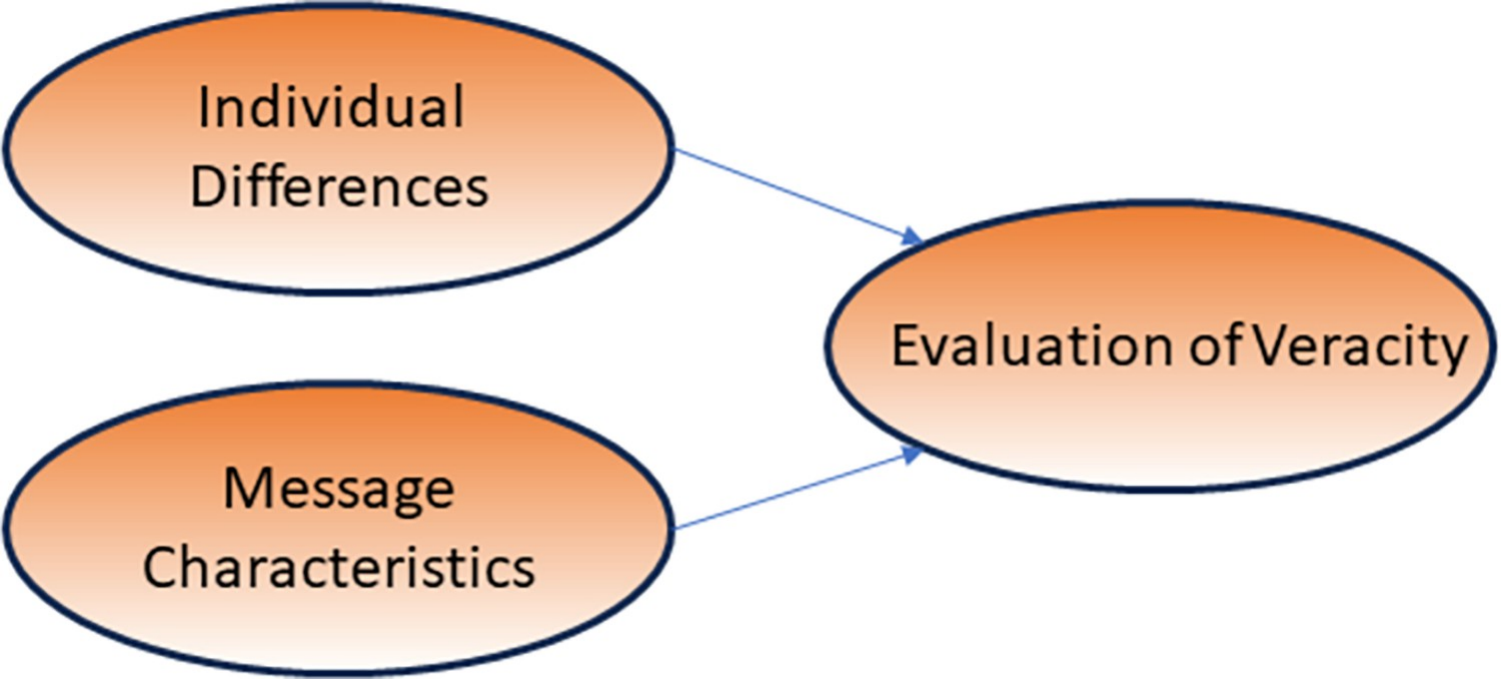
Research model illustrating antecedents to evaluating disinformation veracity.

The reviewed findings build a strong case that both individual differences and specific characteristics of the message, conveyed via social media, influence what people believe, although individual differences may be more important than characteristics of the message. Individual differences that have been shown to affect assessments of credibility include gender, age, education, and need for cognition. Relevant characteristics of the message include source, channel, beliefs about media, familiarity with the story, and how the story is presented (soft vs hard). The respective roles of individual differences and message characeristics are captured in the research model in Fig 1.

## Methods

### Procedure

The study procedures were reviewed and approved by the Iowa State University Institutional Review Board for research involving human subjects (21–456). Because the study was determined to be exempt, consent was obtained orally by the author, who administered all experimental procedures. The author recruited participants in person, by visiting two upper-level management information systems courses in a large business school. The author made a pitch for participation, mentioning the time commitment needed, the nature of the experimental task, and the compensation of $20 USD. Also, the author explained that only those 18 years old or older could take part. Students who were interested in taking part in the study were urged to contact the author, who would then schedule them in a neuroscience lab. Eighteen students from the first class agreed to participate, and 11 reported for the study. Seventeen students from the second class agreed to participate, and 15 reported for the study. In all, 26 participants completed the study. The respondents represent a convenience sample of college students, young and educated, and apparently healthy. The narrowness of the sample will affect the extent to which the findings can be generalized.

Upon entering the lab, each participant sat down in front of a laptop computer which had a GazePoint GP3 eye tracker (60 Hz) attached. The participant then observed 20 social media posts, about 10 different healthcare related topics, one at a time. They were asked to view each post and determine if it was honest or dishonest, true or false, real or fake. They were then asked to describe their reasons for thinking so. Instead of typing, they were asked to vocalize their evaluations and rationale. All of this was captured by the laptop, using GazePoint’s analysis software. Participants were exposed to each post for 30 seconds. The images of the posts advanced automatically. While they worked, the eye tracker captured the motion of their eyes. The eyes of all participants were calibrated for the eye tracker before data collection began. There were no issues with the calibration. However, the final participant started to squint after viewing five or so posts. He was allowed to continue with the experimental task, as his vocalizations could still be used for analysis, even if his eye movements could not be.

The author conducted each session and was present for its entirety. As the participant worked, he watched their eye movements on a different laptop. The author was able to tell if the eye tracker was not able to track a participant’s eyes, and he asked them to correct their position if this occurred. Otherwise, he did not speak. He was seated around the corner from the participant, where he could not be seen.

When they were done, participants completed a paper version of the post-session questionnaire. All participants completed the paper questionnaire, and all answered an attention item correctly. They were then paid $20 USD. The author answered any questions the participants had before they left, and they were later debriefed by email after all data had been collected.

The data captured by the GazePoint software, the transcriptions of participant vocalizations, and questionnaire data were entered into an Excel spreadsheet [63]. All data were cleaned and converted into an SPSS data file. The final N was 520 evaluations (26 * 20), as the object of analysis was the evaluation of the social media post, not the individual participant.

### Experimental stimuli

The purpose of this study was to investigate disinformation about healthcare in social media. While that domain included posts about COVID-19, it was not limited to COVID-19. The author searched the Web to find complementary pairs of social media posts about 10 different healthcare issues: weight loss; cold remedies; diet supplements for muscle gain; chlorine dioxide as a prophylactic and as a cure; measles, mumps, and rubella (MMR) vaccine; diabetes-related diet supplements; antibiotics and viruses; ivermectin as a COVID cure; hydroxychloroquine as a COVID cure; and COVID-19 vaccines in general. Rather than follow the typical research design of deception detection studies, where half of the stimuli are false and the other half true [64], 60% of the posts were chosen to be false. The dishonesty of each false post was certified by articles and warnings published by the U.S. Food & Drug Administration, the U.S. Centers for Disease Control and Prevention, Johns Hopkins Medicine, VaccineWorks, Columbia University Irving Medical Center, and the *New York Times* (S1 Appendix).

### Post-session questionnaire

The post-session questionnaire measured news media literacy, attitudes toward social media, risk propensity, and demographics (S2 Appendix). All scales used had been previously developed and validated in the literature but not in the same study. There were two news media literacy scales that were used: 1) questions about automatic vs mindful thought processing (five items), and 2) questions about media locus of control (six items) [65]. The first scale measures a construct that is similar to need for cognition, according to the scale developers, but within a news media context. The social media scale (named the Social Media Disinformation Scale or SMDS-12) measures beliefs about social media and has three subscales, four items each: 1) consumption, 2) confidence, and 3) sharing [66]. These five scales were measured on a 5-point Likert scale, from 1 for ‘strongly agree’ to 5 for ‘strongly disagree.’ The seven-item scale for risk propensity was developed by Meertens and Lion [67]. The risk propensity scale used a 9-point Likert scale, ranging from 1 for ‘totally disagree’ to 9 for ‘totally agree,’ for its first six items. The seventh item used a 9-point scale, ranging from 1 for ‘risk avoider’ to 9 for ‘risk seeker.’ The four demographic measures used originated in [65]: age, gender (male, female, non-binary, prefer not to say), ethnicity (8 choices), and highest level of school a parent had completed (7 choices).

### Questionnaire confirmatory factor analysis

Each of the three scales was tested with confirmatory factor analysis (CFA), using the AMOS structural equation modeling (SEM) module in IBM’s SPSS statistical package. CFAs were run because all three scales were established and validated elsewhere. Whereas exploratory factor analysis has few metrics to measure validity beyond Cronbach’s alpha, SEM-based CFA analysis provides several goodness-of-fit indices, which provide additional information about the validity of the scales. The details about the CFA process and results are contained in S3 Appendix. None of the scales performed as expected. In each case, a complete model including all items in the scale was run, and this was followed by another model test after items were removed for poor loadings. For the SMDS-12 scale, which had three subscales, the consumption scale did not hold together. A revised model with three items for the confidence subscale and four for the sharing subscale was a very good fit to the data. The average variance explained (AVE) measure and Cronbach’s alpha were also good for both subscales, so they were both retained. The two media literacy subscales had similar problems. Two of the five items for automatic vs. mindful thought processing were dropped for the second CFA, as were three items for the media locus of control subscale. Goodness-of-fit indices improved but were still not adequate. However, the AVE measure and Cronbach’s alpha were excellent for the thoughtful processing subscale, so it was retained. The locus of control subscale was not retained, and it seems likely its poor psychometric qualities contributed to the CFA results for the larger scale. Finally, the risk propensity scale was tested with an initial CFA, and three items were dropped due to poor loadings. The second CFA resulted in three of four adequate goodness-of-fit values, and adequate AVE and alpha values, so a four-item scale was retained.

### Coding responses to open-ended questions

As part of the study procedure, respondents were asked to view social media posts and evaluate their veracity. They were then asked to describe the reasoning that supported their evaluations. Participant vocalizations were all transcribed by the same transcriptionist. Analyzing the similarities and the differences in the reasons that were provided required that the large number of responses be reduced to a smaller and tractable set of codes. The codes used in this study to categorize verbal evaluations were created by the author for a prior study that used the same research stimuli, which participants evaluated and provided the reasons for their decisions. Creating the codes started with reading, analyzing, and categorizing participant responses, and 62 different codes emerged (S4 Appendix). These codes were then applied to all of the participant responses in this prior study. Once this process was complete, 100 records were drawn at random from the data and given to a second coder to check. Initial agreement between the coders on those 100 records was 55%. After several meetings to discuss the discrepancies, changes were made, and the agreement rate rose to 74%. The first coder then corrected errors uncovered during the review exercise for all of the qualitative data. For this study, the author carefully read and analyzed all 520 open-ended responses and applied the same set of 62 codes. The process resulted in the assignment of 656 instances of the codes, as some participants did not provide any reasons, while others provided multiple justifications.

### Analyzing eye tracking data through areas of interest

**Fig 2:**
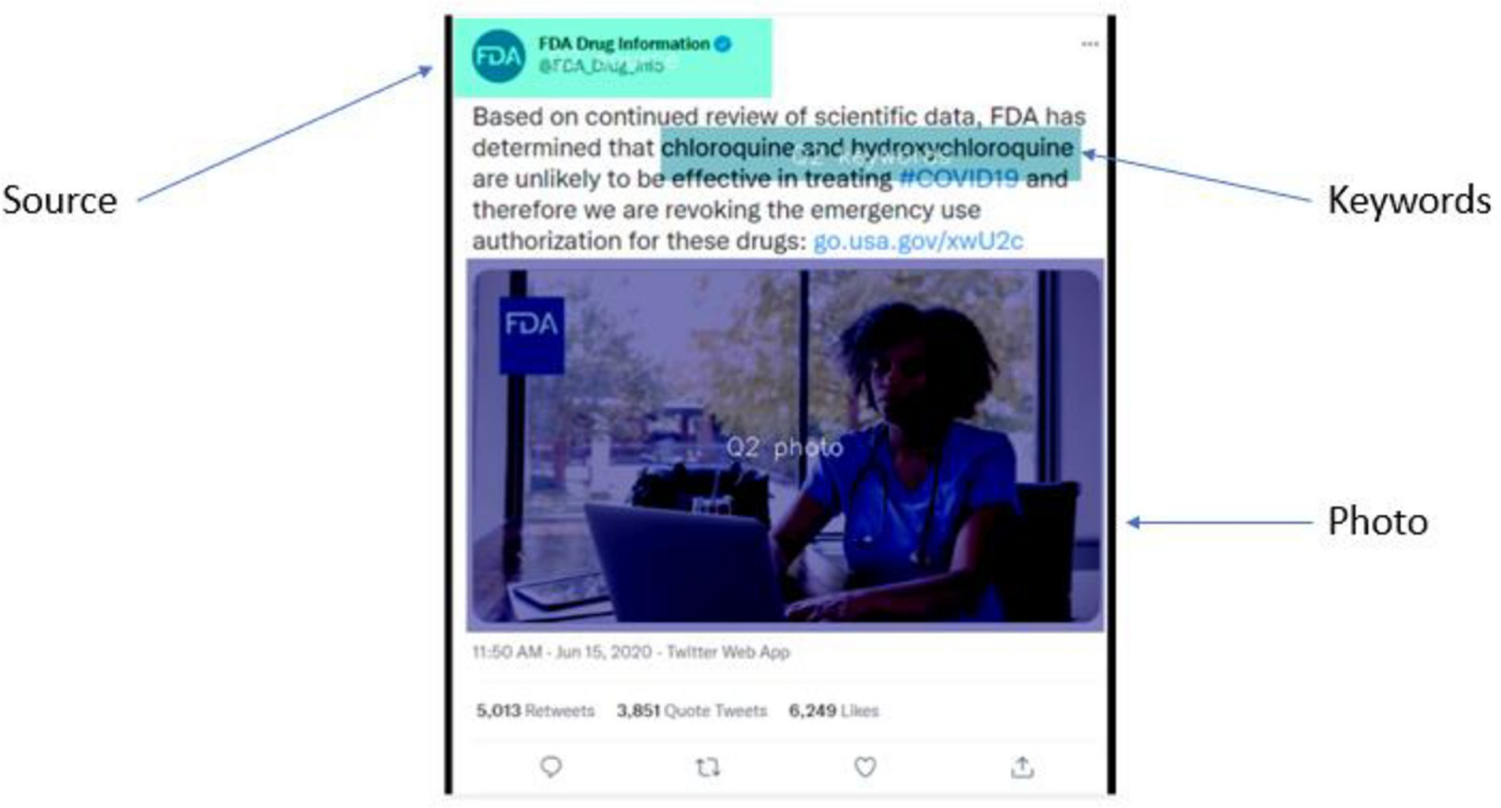
An example of Areas of Interest (AOIs) used in this study.

The common procedure for data collected through eye trackers to be analyzed is by the creation of “Areas of Interest” (AOIs), by which particular areas of an experimental stimulus are defined and drawn [68]. Only some parts of the overall stimulus are of interest to researchers, and designating AOIs allows these parts to be isolated for comparative study. Three AOIs were designated: sources, key words, and photos. The GazePoint analysis software allows researchers to easily draw AOIs, either as rectangles or ovals, and to name and save the AOIs (Fig 2).

The gaze behavior of all participants can then be compared on each AOI. Although there are several metrics for analyzing gaze behavior, the most common metrics are duration of fixations, number of fixations, and number of revisits to an AOI [69, 70].

## Results

The 26 student participants included 18 men and 8 women. They ranged in age from 20 to 26 years; 81% were white; and 89% had a parent with some college education, a college degree, or an advanced degree.

Data analysis covers five different topics: 1) participant success rates for correctly identifying false social media posts; 2) the relationship between individual differences and success at correctly identifying whether a post was true or false; 3) the reasons given by the participants for determining if a post was true or false; 4) the identification of Areas Of Interest (AOI) in the eye tracking data; 5) and the relationships between AOIs as independent variables and success at correctly identifying whether a post was true or false.

### Success rates, overall and by choice

Participants successfully identified the true nature of 69% of the posts (there were 30 instances of posts that were not evaluated as either honest or dishonest). For the eight honest posts, 82% were correctly identified. For the 12 dishonest posts, 61% were correctly identified. This difference is statistically significant: participants were more likely to correctly identify a post as honest when it was honest, and less likely to identify a post as dishonest when it was dishonest (Wald chi-square = 19.867, df = 1, p = .000). Participants correctly identified one post 100% of the time (an FDA post on chlorine dioxide), so this post could not be used for further analysis. Table 1 lists the detection success rates for all 20 posts.

**Table 1:** Detection success rates for all 20 social media posts.

Post	Nature	Special Attributes	Correct %
COVID Vaccine Luciferase	False		86%
FDA Hydroxychloroquine	True		92%
Intermittent Fasting	False		69%
Bogus Cold Remedy	False	No photo	65%
COVID Drug Cocktails	False		65%
FDA Chlorine Dioxide warning	True		100%
Ivermectin as COVID cure	False	No source or photo	73%
FDA Ivermectin warning	True		81%
Dr Rivera’s Chlorine Dioxide cure	False	No photo	65%
Berry Gen treatment for diabetes	False		39%
Nitro Muscle Builder	False		77%
COVID Vaccine opinions by Ben Carson	False		54%
Nyquil Cold Remedy	True	No source	89%
Busch Pro-MMR Vaccine	True	No photo	46%
Antibiotics to Treat COVID	False	No photo	92%
No Antibiotics for Viruses	True		96%
RFK Anti-MMR	False		31%
FDA Semaglutide (Weight Loss)	True		58%
Lysulin treatment for diabetes	False		19%
Harvard Public Health on Supplements	True		88%

### Individual differences with correct as dependent variable

The individual differences variables were tested as independent variables on whether or not participants’ evaluations of true/false were correct, using the MIXED models procedure in SPSS. First, gender was tested and found to have no effect on correctness. The four other individual differences variables were then tested. Only mindful vs. automatic thought processing had a statistically significant effect on correctness, such that more mindful processing (or a greater need for cognition) was associated with correct determinations of the truthfulness of a post (F(1,91) = 7.54, p = .007).

### Reasons for choices

Participants used 47 different reasons to justify their determinations of true or false. They cited these reasons 656 times. Table 2 lists the twenty most listed reasons, which together account for 86% of all justifications. Overall, the source of the post (e.g., FDA, reliable/credible source, reliable article, doctor, Harvard, CDC), whether its claims seemed reasonable (e.g., looks possible/promising; unsubstantiated claims), and the look and feel of the post (e.g., looks fake, biased ad, obvious falsehood), were the most important reasons for determining whether a post was true or false.

**Table 2:** Twenty most cited reasons for judging a post to be true or false.

Reason	Frequency
Looks Possible/Promising	9.2%
FDA	9.0%
Verified	6.9%
Unsubstantiated Claims	6.7%
False Post Looks Fake	6.6%
False Post is Biased Ad	5.0%
Obvious Falsehood	4.3%
False Post Has No Credentials	4.3%
Cannot Decide	4.1%
False Post Based on Opinion	3.4%
Links	3.2%
False Post from Random Person	3.2%
Reliable Article	3.1%
Reliable/Credible Source	3.1%
Doctor	2.9%
Familiarity	2.9%
Harvard	2.7%
CDC	2.3%
Photo	2.0%
Antibiotics Are Not for Viruses	1.7%
TOTAL	86.3%

While the meanings of source and reasonable health claims are fairly evident, it is useful to explore in more detail the meaning of “look and feel” in this context. As would be expected, many participants cited the photos in posts (e.g., “names on the actual picture itself,” “the visual kind of gives it away and it just seems a little sketchy,” “They just took two pictures of Jennifer Aniston and put them next to each other”). Other participants listed specific elements of the posts that were not related to photos (“I think that’s false because she has an emoji by her name, first of all. Just the vocab she’s using,” “it just looks fake–I guess like the spelling too,” “The way it looks and the–yeah–the color and everything,” “that’s why there’s the bright colors,” “Lots of variations in the typing, using short apostrophes, emojis, capitalizations”). Others had more holistic evaluations (e.g., “doesn’t look super legit,” “it just feels kind of, like, dubious,” “propaganda–they can put whatever they want on there,” “it’s conspiracy propaganda,” “crazy marketing,” “them just saying that the glucose reacts with the blah, blah, blah, all of this stuff,” “it looks like something I would see on TV at three in the morning”). When justifying their conclusions that the posts were obvious falsehoods, participants tended to cite some of the claims being made (e.g., “Right off the bat, I just feel like this is completely false–I don’t think it’s really possible for something like that to happen,” “I mean, come on, like chlorine dioxide, I believe that would probably kill someone,” “I would not trust this because there’s no way hospitals are getting paid, like they want to save people–they don’t want people to die,” “I don’t think any person of the US Embassy would question the MMR vaccine”). “Look and feel,” then, included both photographic elements and other visual elements, such as emojis and colors, as well as holistic evaluations and content that rendered a post obviously false.

Dividing the posts into sets of true (N = 8) and false (N = 12) posts revealed that participants relied on different patterns of reasoning for true posts and for false ones, implying that the nature of a post inspired different strategies for making veracity judgments. Table 3 compares the top fifteen reasons given for veracity judgments, when the posts are either true or false. There were 36 different reasons given for a true post, for a total of 296 responses. There were 37 different reasons given for a false post, for a total of 360 responses. Seven reasons are listed for both true and false posts, so there is some overlap in how posts were judged. However, these seven reasons account for almost twice as much of the judgment of false posts (40.3%) as of true posts (22.3%). When evaluating true posts only, source was the most important factor. When evaluating false posts only, the nature of the claims being made, and the look and feel of the post were most important (see ‘design look,’ [11]).

**Table 3:** Comparing reasons for judgment based on the nature of the post.

True Posts		False Posts	
Reason	Frequency	Reason	Frequency
FDA	20.0%	Looks Possible/Promising	12.8%
Verified	15.2%	Unsubstantiated Claims	11.4%
Links	6.8%	False Post Looks Fake	9.7%
Harvard	6.1%	False Post is Biased Ad	8.3%
Familiarity	5.4%	Obvious Falsehood	7.2%
CDC	4.7%	False Post Has No Credentials	6.4%
Looks Possible/Promising	4.7%	Cannot Decide	6.1%
Reliable/Credible Source	4.4%	False Post from Random Person	5.3%
Reliable Article	3.4%	False Post Based on Opinion	4.2%
Antibiotics Are Not for Viruses	3.4%	Photo	3.1%
Doctor	3.0%	Reliable Ad	2.8%
False Post Looks Fake	2.7%	Reliable Article	2.8%
False Post Based on Opinion	2.4%	Doctor	2.8%
Cannot Decide	1.7%	Reliable/Credible Source	1.9%
Claims Can Be Proven	1.7%	False Post Unprofessional	1.9%
TOTAL	85.5%	TOTAL	86.7%

### Areas of interest as independent variables and ‘correct’ as dependent variable

The three metrics that were used in this study for each AOI were duration of fixations, number of fixations, and number of revisits to an AOI [68, 69]. However, as the data were not normally distributed, they were transformed using a square root transformation. Not every participant fixated on every AOI, meaning their values for that AOI were zero. A square root transformation, as opposed to other possible transformations, allows those zero values to remain in the data. In total, then, there were nine AOI measures: total fixation time, total number of fixations, and total number of revisits to an AOI, for each AOI (key words, sources, and photos).

In the analysis, AOI measures were treated as independent variables, with Correct as the dependent variable. The MIXED models procedure in SPSS was used for the analysis, due to the repeated measures nature of the data. One participant had to be dropped from the analysis, as he squinted through much of the experimental session. This left an N of 500 evaluations of social media posts. Also, not every post could be used in every analysis. The post where participants were 100% successful in their evaluations (the FDA post on chlorine dioxide) had to be dropped due to a lack of variance. This left 19 posts with key words, or an N of 475 (e.g., 25 * 19), which could all be used to test the effects of the key words AOI measures. Next, two posts lacked clear sources (posts on ivermectin as a COVID cure and about Nyquil), so once they were dropped, that left 17 posts where the AOIs for key words and sources could be analyzed (N = 425 posts). Finally, five posts lacked photos (posts promoting a bogus cold remedy, chlorine dioxide, the MMR vaccine, and using antibiotics to treat the COVID virus; the pro-ivermectin post also lacked a clear source and had already been excluded), and once dropped, that left 13 posts which could be analyzed using all nine AOI measures, for key words, sources, and photos (N = 325). Table 1 shows which of the posts used in each set of analyses were honest or dishonest. The set of 19 consisted of seven honest and 12 dishonest posts; the set of 17 consisted of six honest and 11 dishonest posts; the set of 13 consisted of five honest and eight dishonest posts.

The effects of key words on the correct evaluation of social media posts were tested first, on the 19 posts that contained key words. Both the total fixation time on key words (F(1,66) = 19.25, p < .000) and the total number of fixations on key words (F(1,42) = 19.43, p = .001) were statistically significant. However, the findings pointed in different directions: The more total view time of key words, the more likely the assessment of the post was incorrect; the more total fixations on keywords, the more likely the assessment was correct. Revisits to the key word AOI were not significant.

The two posts without clear sources were then dropped, and the analysis on Correct was run again, with the six AOI measures for key words and sources. Once again, both total view time of key words (F(1,107) = 24.74, p < .000) and number of fixations on key words (F(1, 133) = 22.93, p = .009) were statistically significant. The directions of the relationships were the same: more viewing time was associated with an incorrect assessment, while more fixations were associated with a correct assessment. The relationship between revisits to keywords and correct assessments was not statistically significant. The total view time for sources was not statistically significant, but the total number of fixations on sources (F(1,233) = 5.28, p = .023) was. More fixations on sources were associated with an incorrect assessment. Also, more revisits to sources was statistically significant (F(1,290) = 6.06, p = .014), such that more revisits to sources were associated with correct assessments.

Finally, four more posts were dropped from the analysis, as they lacked photos. The evaluations of the remaining 13 posts were analyzed for the nine AOI measures for key words, sources, and photos. As with the previous two analyses, total viewing time of key words (F(1,83) = 15.07, p = .000) and number of fixations on key words (F(1, 102) = 12.83, p = .001) were both significant. More viewing time of key words led to incorrect assessments, while more fixations led to correct assessments. Similarly, the relationships between the number of fixations on sources (F(1,188) = 4.69, p = .032) and revisits to sources (F(1,188) = 4.70, p = .031) were significant. More fixations on sources were associated with incorrect assessments, and more revisits to sources were associated with correct assessments. No other AOI measures were statistically significant. (For complete statistical results, see S5 Appendix.) For this last comparison, where participants were able to view keywords, sources, and photos, they spent an average of 13.58 seconds, or 45% of the 30 seconds allotted, fixating within the AOIs. Of that time, they spent 5.5 seconds fixating on keywords, 3.6 seconds fixating on sources, and 4.5 seconds fixating on photos.

## Discussion

The purpose of this study was to discover factors that influenced how people evaluated the veracity of disinformation about healthcare. The study was inductive, designed to gather data through controlled observation. The findings can be used to form empirical generalizations, which can then be used to inform theory development. Participants were reasonably successful, with a 69% success rate, at detecting disinformation in healthcare social media posts. This success rate is higher–by 15 percentage points—than the recognized success rate for the detection of deceptive communication generally, at 54% [63], and it is consistent with a past finding on disinformation detection [56]. Success rates varied from a low of 19% (Lysulin) to complete agreement (100%) (FDA post on chlorine dioxide).

The study looked at individual differences and characteristics of the posts, given that the relevant literature focused on these factors as helping to explain disinformation detection success. One individual difference variable was found to be related to how participants viewed the posts. Participants who espoused a belief in mindful processing (or a need for cognition) were more successful than those who espoused a belief in automatic processing. The relationship between need for cognition and skepticism about disinformation was demonstrated in earlier studies [32, 42].

As for the characteristics of a post, in general, the most cited factors for determining if a post was true or false were source, whether the claims being made were seen as reasonable, and “look and feel.” For true posts, 53% of the reasons cited dealt with source. For false posts, 22% of the reasons were about source (no credentials, random person, reliable ad, reliable article, doctor, reliable/credible source), while 27% of the reasons were related to the “look and feel” of false posts (looks fake, is biased, obvious falsehood, unprofessional). These findings point to the prominence of source and design “look and feel” when determining if a social media post about healthcare is true or false. Although past research has emphasized the importance of “look and feel” [16], support for the importance of source is mixed [29, 40, 42, 44–46]. However, in all of these studies, the sources and posts that made up the experimental stimuli were simulated, while the sources and posts used in this study were real.

The characteristics which attracted the visual attention of participants were also evaluated through “Areas of Interest” analysis. Three different AOIs were identified and used in the analysis: key words, source, and photos. Given the 30 seconds they were allowed to examine each post, participants on average spent only a few seconds fixating within each AOI: 5.5 seconds on keywords, 3.6 seconds on sources, and 4.5 seconds on photos. Their rapid processing speeds lend support to viewing social media assessment through the lens of dual processing theories such as ELM [50]. Participants seem to have engaged in rapid heuristic-based processing of the content of each social media post. Their behavior supports the calls for additional research of disinformation from the perspective of dual processing theories and for increasing the use of eye tracking technology in such studies [28, 30].

Across three analyses where AOIs were independent variables and the dependent variable was detection success, two factors were always statistically significant: the total view time of key words, and the total number of fixations on key words. More time viewing key words led to incorrect assessments, while more fixations led to correct assessments. Apparently spending a large amount of time studying key words can be misleading when looking for cues to disinformation, but looking at key words multiple times is associated with correct assessments. In the second and third analyses, when sources and photos respectively were added, two additional factors were statistically significant: the total number of fixations on sources, and the total number of revisits to sources. More fixations on sources were associated with an incorrect assessment, and more revisits to sources were associated with correct assessments. The results do seem to show that total fixation duration is a distinct construct from the number of fixations, and the number of fixations is distinct from the number of revisits, given the divergence in findings. It also seems to be the case that words alone, as in key words, are evaluated differently from the evaluations of how sources are represented on social media, which often include thumbnail photos, handles, and in the case of Twitter, indicators of authenticity. Key words seem to require multiple viewing to decode their value in detecting disinformation, while the richer source indicators require more viewing time. This interpretation is, of course, speculative, at this point. Interestingly, a large number of fixations on a social media post object is not a guarantee of success in detecting disinformation. It depends on what the object is. More fixations on key words were associated with better assessments, but more fixations on sources were associated with worse assessments of credibility. In addition, in the third analysis, none of the AOIs related to photos had any effect on detection success. To summarize, there is support for associations between more fixations on keywords, and more revisits to sources, and successfully detecting disinformation.

As reported previously, when evaluating whether or not a post was true/honest/real, the reasons participants relied on differed if the post was true or if it was false. Participants were better able to successfully detect true posts than false posts.

### Implications, limitations and future research

The findings from this study are relevant to both practice and research. For practice, it helps establish a baseline for expectations about how successful people can be at detecting health-related disinformation and how to move forward from that baseline. If college students on average were successful at detecting the truth or falsity of 69% of the posts they were exposed to in this study, then proactive or corrective behavior can focus on posts similar to the 31% they got wrong. From Table 1, the posts with the lowest success rates dealt with purported cures for diabetes (Lysulin and Berry Gen), two posts (one false and one true) about the MMR vaccine, one about a politician’s attempts to gain from attempts to discredit COVID-19 vaccines, and one post about the potential for semaglutide for weight loss. It would seem, based on these findings, that more attention could paid to how to interpret ads for health products with dubious claims, and how to interpret information posted about vaccines in general and the MMR vaccine in particular. While the post about semaglutide was honest, and only 58% of participants recognized this, the finding may reflect a healthy skepticism on the part of those who decided the claims about weight loss were not believable. The post in question was actually a repost of a certified FDA post, and the FDA was considered by respondents to be very credible in every other post where the agency’s name appeared in the experimental stimuli. In the case of “miracle” weight loss drugs, however, the subject matter may have trumped the source.

The findings also have implications for research about successfully detecting disinformation about healthcare. The findings add to our knowledge of how well people can detect disinformation, supplementing what is known about how well people can detect deceptive communication in general. Future research can investigate the differences between general deceptive communication and healthcare-related disinformation to understand why people are better at the latter than at the former. The findings regarding the factors people rely on to successfully detect disinformation can form the basis for generalizations about the factors that people take into account when evaluating disinformation in social media. These generalizations can then become the basis for theories about disinformation and its successful detection.

These findings, together with those from two other eye tracking studies [28, 30] suggest that dual processing theories may be used to help better explain the manner in which people evaluate the veracity of social media posts about healthcare. Given how quickly people process social media posts, it seems they tend to rely on heuristics to evaluate veracity. The common response of “look and feel” for the rationale behind their veracity decisions illustrates the lack of in-depth processing. However, we do not know the time threshold for moving from a heuristic approach to an analytical one. Between-subject laboratory experiments that vary the time available to participants, say from 10 seconds per post to one minute or more, may discover the point at which participants begin to move beyond heuristics. It is an open question as to whether more time for evaluation actually leads to more successful detection of disinformation. Such studies should be designed with eye tracking and rationale self-reporting as part of the research protocol.

As is the case with all research, this research has limitations. The participants were all American college students, and most of them were white, young, and from well-educated families. Their youth and their likely lack of exposure to serious illnesses no doubt influenced their ability to accurately detect healthcare related disinformation. A less healthy sample would likely have had stronger motivations for detecting disinformation and a better experience base to rely on in their evaluations. Similarly, two of the false posts contained claims about drugs that could mitigate or even cure a very specific disease, diabetes. Respondents who were not diabetic or who had little knowledge of the disease and how it works would have been at a disadvantage in their attempts at detecting disinformation. On the other hand, at the time the data were collected, the COVID-19 pandemic was still recent and still uppermost in many people’s minds. No doubt the participants had been exposed to a large amount of information about the virus and its treatment, and this familiarity may have influenced their evaluations of posts related to COVID-19. Also, the posts used in this study were actual posts from social media, chosen to increase their external validity and salience to participants. Because of this design decision, some posts may have included more text and more keywords than others (although efforts were made to keep the number of keywords as constant as possible across posts), and this variability may have influenced the total duration of fixations and the number of fixations on keywords. A study with manufactured posts that controlled the number of keywords, the amount of text, and the size and content of photos could be designed to test their relative roles in the evaluation of social media posts for disinformation. Other studies could focus on a different set of areas of interest than the ones used here.

Another limitation is that the order in which social media posts were presented to participants was not varied. Although potentially risky, this was intentional. Sixty percent of the posts were false, so in any possible order, some of the false posts would appear in clusters. Similarly, the 20 posts consisted of 10 pairs about 10 different topics, but in the static ordering, only one pair appeared together by topic (numbers 15 and 16). The static order of presentation was designed to reduce the chances of spurious findings due to large runs by the nature of the post or multiple groupings by the topic of the post. Still, the static order could have resulted in a familiarity effect or a fatigue effect. An examination of the order of presentation and participant success rates for detecting disinformation (Table 1) shows that neither of these effects occurred. Instead, as reported previously, a key variable related to detection success was the nature of the post. The overall success rate was 69%, but it was 82% for true posts and 61% for false posts. Based on past research in the deception literature [71], this finding was expected. People have been shown to be better at detecting true statements than they are at detecting false statements. The order of presentation did not affect the relationship between the nature of the post and known patterns of detection success. Controlling for order, the partial correlation between the nature of the post and detection success was -.263 (2-tailed, p < .000). (The negative correlation is due to how success and the nature of the post were coded: 1 for correct and 2 for incorrect evaluations; 1 for false posts and 2 for true posts.) In short, while the presentation order may have influenced detection success, there is evidence that the nature of the post had more of an impact.

In addition, this study could be replicated with other groups to determine how those who are less educated or less healthy or those who are not young Americans would fare in attempting to successfully detect disinformation about healthcare. The findings might differ as well in countries that have nationalized healthcare systems or other types of social systems that differ from those found in the U.S. The findings might also differ across cultures generally. Finally, not all of the factors associated with believing or not believing disinformation were included in this study. For example, neither the emotional content of the posts nor the emotional state of the viewer were considered. These, and potentially other important factors, could be added to future research.

Finally, it is important to note that the worlds of social media and healthcare disinformation are not static. In the time since the data reported on here were collected, in early 2022, Twitter and its verifying blue check mark ceased to exist, at least as they were known before the purchase of Twitter by Elon Musk in October 2022. Similarly, although COVID-19 has not been eradicated and continues to mutate and spread, it is no longer as uppermost in the public’s mind in August 2023 (when this was written) as it was in early 2022 (when the data were collected). Were this study to be replicated today, it is an open question whether the same results would follow, given these and other changes in the social media and healthcare disinformation landscape.

## Conclusion

Disinformation, about various topics, continues to be a force across social media. The primary reason for conducting this study was to determine the ubiquity of belief in health-related disinformation and to better understand the factors that led to that belief. Study participants were able to successfully determine the honesty or dishonesty of a post 69% of the time. The primary factors they relied on to make their judgments were the source of the post, the veracity of the claims being made, and the design look and feel of the post. Eye tracking analysis showed the importance of keywords and source to making appropriate judgments. The findings suggest the outlines of generalizations that can be made about why people believe online disinformation, but much work remains to be done to form the basis for a more complete mid-range theory.

## Supporting information

S1 Appendix Documentary evidence of disinformation in posts considered false. (DOCX)

S2 Appendix Survey instrument. (DOCX)

S3 Appendix CFA process and results. (DOCX)

S4 Appendix Codes derived from responses to open-ended questions about rationale. (DOCX)

S5 Appendix Additional data analysis results. (DOCX)
