# Supplementary material for: Discovering why people believe disinformation about healthcare
Source: PLoS One. 2024 Mar 21;19(3):e0300497. doi: 10.1371/journal.pone.0300497 (PMC10956743; doi:10.1371/journal.pone.0300497)
Supplement: S1 Appendix — (DOCX) [file pone.0300497.s001.docx]

**Documentary Evidence of Disinformation in Posts Considered False**

The study utilized 20 social media posts (or similar), 12 of which were considered false/dishonest and 8 of which were considered true/honest. The basis for considering these posts false/dishonest came from the sources listed in the text: U.S. Food & Drug Administration, the U.S. Centers for Disease Control and Prevention, Johns Hopkins Medicine, VaccineWorks, Columbia University Irving Medical Center, and the *New York Times.* Links to the documentary evidence for each of the 12 false/dishonest posts are listed below, along with brief overviews of the content of the evidence.

1 & 2) Lysulin and Berry Gen:

“FDA and FTC Send Warning Letters to 10 Companies for Illegally Selling Dietary Supplements Claiming to Treat Diabetes”

Lysulin and Berry Gen have not been evaluated by the FDA to be safe and effective.

FDA announcement, 9/9/21: <https://www.fda.gov/food/cfsan-constituent-updates/fda-and-ftc-send-warning-letters-10-companies-illegally-selling-dietary-supplements-claiming-treat>

3) Intermittent Fasting:

A study from the University of California San Francisco found that intermittent fasting does lead to weight loss, but it is due more to the loss of muscle than of fat.

O’Connor, A. 2020. A Potential Downside to Intermittent Fasting. Sept 28, 2020. NYTimes. <https://www.nytimes.com/2020/09/28/well/eat/a-potential-downside-of-intermittent-fasting.html>

4) Ivermectin:

A large study in Brazil found that ivermectin does not successfully treat COVID-19.

Zimmer, C. 2022. Ivermectin Does Note Reduce Risk of COVID Hospitalization, Large Study Finds. March 30, 2022. NYTimes. <https://www.nytimes.com/2022/03/30/health/covid-ivermectin-hospitalization.html>

5) Hydroxyclorquine:

“A study of nearly 1,400 patients with moderate to severe COVID-19 disease at a single New York hospital found that patients who received the drug fared no better than patients who did not receive the drug.”

Columbia University Irving Medical Center. 2020. Hydroxychloroquine: First Large Study Does Not Support Routine Use in COVID-19 Patients. May 7, 2020. <https://www.cuimc.columbia.edu/news/hydroxychloroquine-first-large-study-does-not-support-its-routine-use-covid-19-patients>

6) Muscle gain:

Due to TikTok and other influences, a generation of young men are devoting their time to weightlifting and devouring protein to get a “Dorito” shaped body (broad shoulders, small waist), to the detriment of their education and social skills. They have learned that highly promoted protein powders do not work or are not enough.

Hawgood, A. 2022. What is ‘Bigorexia?’ March 5, 2022. NYTimes. <https://www.nytimes.com/2022/03/05/style/teen-bodybuilding-bigorexia-tiktok.html>

7) MMR:

The CDC report says “One dose of MMR vaccine is 93% effective against measles, 78% effective against mumps, and 97% effective against rubella. Two doses of MMR vaccine are 97% effective against measles and 88% effective against mumps.” These findings counter claims that the MMR vaccine is ineffective.

CDC. 2021. Measles, Mumps, and Rubella (MMR) Vaccination: What Everyone Should Know. <https://www.cdc.gov/vaccines/vpd/mmr/public/index.html>

8) Antibiotics and viruses:

Antibiotics are not recommended for treating viruses, due to the risk of bacterial resistance.

Johns Hopkins Medicine. 2022. Antibiotics. <https://www.hopkinsmedicine.org/health/wellness-and-prevention/antibiotics>

9) COVID vaccine & luciferace:

Luciferase is an enzyme that allows fireflies to glow. Although luciferase was used in some clinical tests of the COVID-19 vaccine, it is not in the vaccine, and it is not injected into humans.

VaccinesWork. 2021. Claims that COVID jabs can be used to track you with ‘luciferase’ are false – the substance isn’t even in the vaccine. <https://www.gavi.org/vaccineswork/claims-covid-jabs-can-be-used-track-you-luciferase-are-false-substance-isnt-even>

10) COVID vaccine as ‘giant experiment:’

The two mRNA vaccines, from Pfizer and Moderna, are safe and good at preventing serious or fatal cases of the COVID-19 variants available in the U.S. through 2022.

Maragakis, L. & Kelen, G.D. 2022. Is the COVID-19 Vaccine Safe? Johns Hopkins Medicine. <https://www.hopkinsmedicine.org/health/conditions-and-diseases/coronavirus/is-the-covid19-vaccine-safe>

11) Chlorine Dioxide:

The FDA has repeatedly warned consumers not to drink chlorine dioxide products sold as miracle cures.

FDA. 2020. Coronavirus (COVID-19) Update: FDA Warns Seller Marketing Dangerous Chlorine Dioxide Products that Claim to Treat or Prevent COVID-19. April 8. 2020. <https://www.fda.gov/news-events/press-announcements/coronavirus-covid-19-update-fda-warns-seller-marketing-dangerous-chlorine-dioxide-products-claim>

12) Cocktail as a Cold Remedy:

This post needs no citation to show it is false, as it is ridiculous on its face. What medical doctor would write a prescription for a cocktail as a cold remedy? Also, other information included in the post is evidence of its lack of candor – for example, the “doctor” who signed the prescription is Dr. Hugo Z. Hackenbush, Grouch Marx’s character in the Marx Brothers film "A Day at the Races."
