# Supplementary material for: Discovering why people believe disinformation about healthcare
Source: PLoS One. 2024 Mar 21;19(3):e0300497. doi: 10.1371/journal.pone.0300497 (PMC10956743; doi:10.1371/journal.pone.0300497)
Supplement: S2 Appendix — (DOCX) [file pone.0300497.s002.docx]

**Please Choose the Best Response.**

|  | **Strongly Agree** | **Agree** | **Neutral** | **Disagree** | **Strongly Disagree** |
| --- | --- | --- | --- | --- | --- |
| **Questions about Automatic vs. Mindful Thought Processing [35]** | | | | | |
| MAC1: I don’t like to have to do a lot of thinking. | O | O | O | O | O |
| MAC2: I try to avoid situations that require thinking in depth about something. | O | O | O | O | O |
| MAC3: I prefer to do something that challenges my thinking abilities rather than something that requires little thought. | O | O | O | O | O |
| MAC4: I prefer complex to simple problems. | O | O | O | O | O |
| MAC5: Thinking hard and for a long time about something gives me little satisfaction. | O | O | O | O | O |
| **Questions about Media Locus of Control [35]** | | | | | |
| MAC6: If I am misinformed by the news media, it is my own behavior that determines how soon I will learn credible information. | O | O | O | O | O |
| MAC7: I am in control of the information I get from the news media. | O | O | O | O | O |
| MAC8: When I am misinformed by the news media, I am to blame. | O | O | O | O | O |
| MAC9: The main thing that affects my knowledge about the world is what I myself do. | O | O | O | O | O |
| MAC10: If I pay attention to different sources of news, I can avoid being misinformed. | O | O | O | O | O |
| MAC11: If I take the right actions, I can stay informed. | O | O | O | O | O |
|  | **Strongly Agree** | **Agree** | **Neutral** | **Disagree** | **Strongly Disagree** |
| **SMDS-12 Scale: Consumption [36]** | | | | | |
| SMDS1: I am looking for news about the coronavirus disease (COVID-19) in social networks. | O | O | O | O | O |
| SMDS2: People who are part of my social networks disseminate information about the coronavirus (COVID-19). | O | O | O | O | O |
| SMDS3: All news regarding the coronavirus disease (COVID-19) is accessible to me from my social networks. | O | O | O | O | O |
| SMDS4: I never worry about finding information about the coronavirus (COVID-19) from social media. | O | O | O | O | O |
| **SMDS-12 Scale: Confidence [36]** | | | | | |
| SMDS5: I trust the information disseminated on social networks about the coronavirus disease (COVID-19). | O | O | O | O | O |
| SMDS6: News on social media about the coronavirus disease (COVID-19) are credible in its majorities. | O | O | O | O | O |
| SMDS7: The information disseminated on social networks about the disease of the coronavirus (COVID-19) is credible to me. | O | O | O | O | O |
| SMDS8: I never trust information posted on social media about the coronavirus disease (COVID-19). | O | O | O | O | O |
|  |  |  |  |  |  |
|  | **Strongly Agree** | **Agree** | **Neutral** | **Disagree** | **Strongly Disagree** |
| **SMDS-12: Sharing [36]** | | | | | |
| SMDS9: I share all coronavirus disease (COVID-19) information on social media. | O | O | O | O | O |
| SMDS10: I choose the information related to the coronavirus disease (COVID-19) that I share. | O | O | O | O | O |
| SMDS 11: I often rebroadcast information I receive about coronavirus disease (COVID-19). | O | O | O | O | O |
| SMDS12: People on my social networks often receive information related to coronavirus disease (COVID-19) from my account. | O | O | O | O | O |

**Risk Propensity Scale [37]**

**Please indicate the extent to which you agree or disagree with the following statement by putting a circle around the option you prefer. Please do not think too long before answering; usually your first inclination is also the best one.**

|  | **Totally Disagree** | **2** | **3** | **4** | **Neutral** | **6** | **7** | **8** | **Totally Agree** |
| --- | --- | --- | --- | --- | --- | --- | --- | --- | --- |
| Risk1: Safety first. | O | O | O | O | O | O | O | O | O |
| Risk2: I do not take risks with my health. | O | O | O | O | O | O | O | O | O |
| Risk3: I prefer to avoid risks | O | O | O | O | O | O | O | O | O |
| Risk4: I take risks regularly. | O | O | O | O | O | O | O | O | O |
| Risk5: I really dislike not knowing what is going to happen. | O | O | O | O | O | O | O | O | O |
| Risk6: I usually view risks as a challenge. | O | O | O | O | O | O | O | O | O |

**Risk7: I view myself as a …**

| **Risk Avoider** | **2** | **3** | **4** | **Neutral** | **6** | **7** | **8** | **Risk Seeker** |
| --- | --- | --- | --- | --- | --- | --- | --- | --- |
| O | O | O | O | O | O | O | O | O |

**What is your gender?**

O Male

O Female

O Non-binary

O Prefer not to say

**What is the year of your birth?**

**What is your ethnicity?**

O White

O African American

O Latino/Hispanic/Latinx

O Pacific Islander

O Native American/First Peoples

O Asian American

O Multiracial

O Other

**What is the highest level of education your mother/father has completed?**

O Less than high school

O High school/GED

O Some college

O Vocational/Technical/Community College degree

O Four-year college degree

O Advanced degree (e.g., MFA, master’s, PhD)

O Don’t know/Not sure
