# Supplementary material for: Discovering why people believe disinformation about healthcare
Source: PLoS One. 2024 Mar 21;19(3):e0300497. doi: 10.1371/journal.pone.0300497 (PMC10956743; doi:10.1371/journal.pone.0300497)

**Appendix: CFA process and test results**

All three scales were subjected to confirmatory factor analysis (CFA), given that each scale is established and has been validated in past research. The scales were analyzed using structural equation modeling with IBM’s AMOS version 29. The result of testing the SMDS scale is shown in Figure S3.1.

**Figure S3.1: Result of SMDS scale CFA test.**


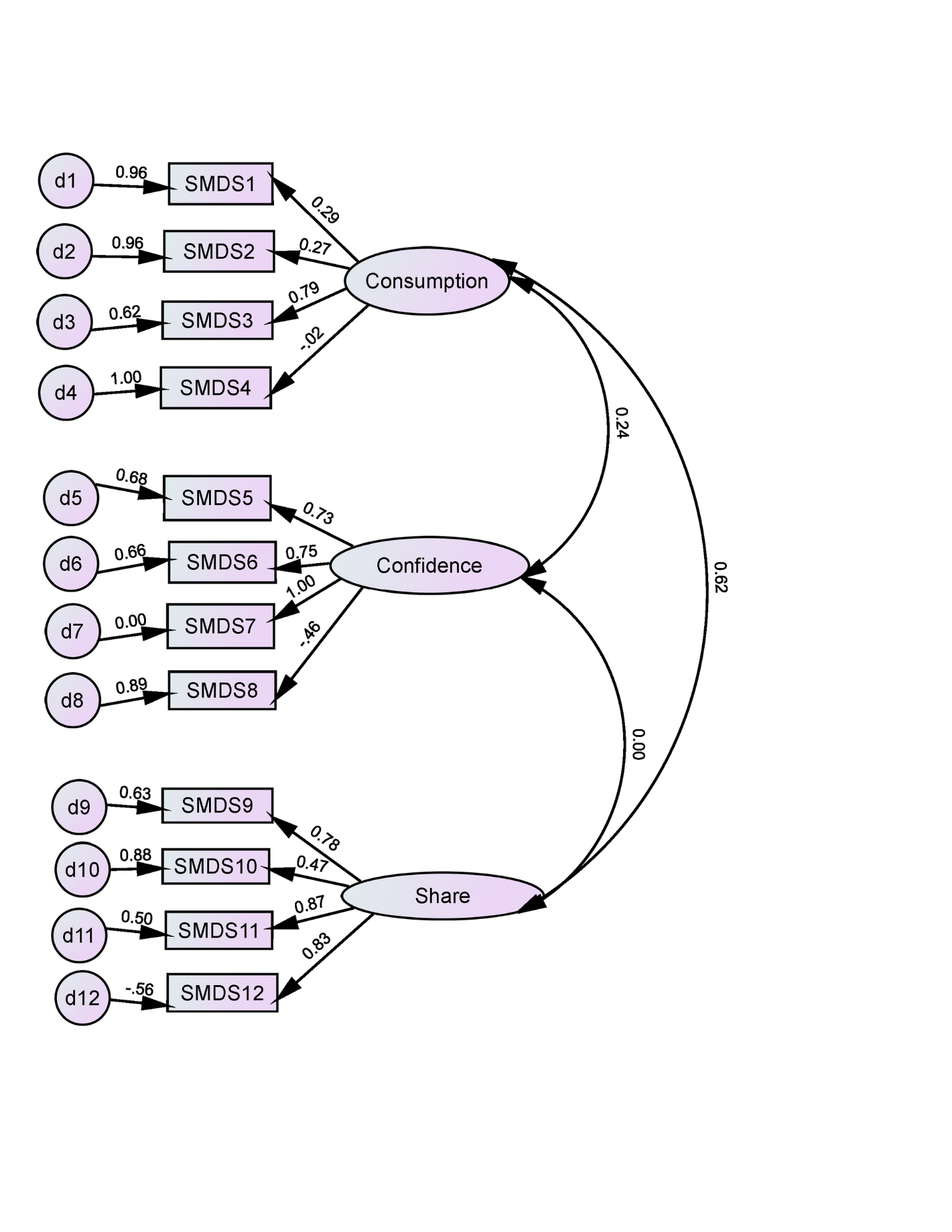


The SMDS scale has three subscales. Two of the subscales, Confidence and Share, held together well, based on the loadings of the items. For Consumption, the loadings for three of its four items were poor. Also, for Confidence, item SMDS8 was negative. Accordingly, all four of the items for Consumption and item SMDS8 were dropped and the CFA was rerun. There were no differences for the item loadings for Confidence. The loadings for Sharing increased. Table S3.1 provides a comparison of kept item loadings for the original and subsequent CFAs. It also shows the internal consistency reliability of the scale, using Cronbach’s alpha test, and the value for average variance explained (AVE). The reliability and AVE values are considered acceptable for both subscales.

**Table S3.1: CFA statistical results for SMDS scale**

|  | **Original CFA** | **Second CFA** | **Internal Consistency**  **Reliability** | **Average**  **Variance**  **Explained** |
| --- | --- | --- | --- | --- |
| **Confidence** |  |  | 0.824 | 0.701 |
| SMDS5 | 0.73 | 0.73 |  |  |
| SMDS6 | 0.75 | 0.75 |  |  |
| SMDS7 | 1.00 | 1.00 |  |  |
| **Share** |  |  | 0.75 | 0.570 |
| SMDS9 | 0.78 | 0.78 |  |  |
| SMDS10 | 0.47 | 0.48 |  |  |
| SMDS11 | 0.87 | 0.87 |  |  |
| SMDS12 | 0.83 | 0.87 |  |  |

Table S3.2 shows the goodness of fit statistics for both CFA tests. All of the indices were better for the second CFA than for the first, implying the second model was a better fit to the data. The Chi-square/df value indicates an acceptable fit, the CFI value indicates an excellent fit, and RMSEA value is close to the 0.05 value, which indicates a reasonable fit.

**Table S3.2: Goodness-of-fit indices for SMDS scale**

| **Fit Index** | **Original CFA** | **Second CFA** |
| --- | --- | --- |
| Chi-square | 70.27 | 14.607 |
| df | 51 | 13 |
| p | .038 | .033 |
| Chi-square/df | 1.37 | 1.12 |
| CFI | 0.811 | 0.979 |
| NFI | 0.581 | 0.849 |
| RMSEA | 0.123 | 0.070 |

The MAC scale has two subscales, Thought Processing (or Mindfulness) and Media Locus of Control (Figure S3.2). The first subscale has five items; the second has six. The results of the first CFA show two items (MAC4 and MAC5R (R for reverse coded)) had very low loadings on Mindfulness. Three of the six items for Locus of Control (MAC6, MAC7, and MAC10) had loadings less than 0.5. For the subsequent CFA, these five items were dropped. Table S3.3 compares the kept item loadings for both subscales. It also contains internal consistency reliability and AVE values. The values for Thought Processing are acceptable, but those for are not.

**Figure S3.2: Result of MAC scale CFA test.**


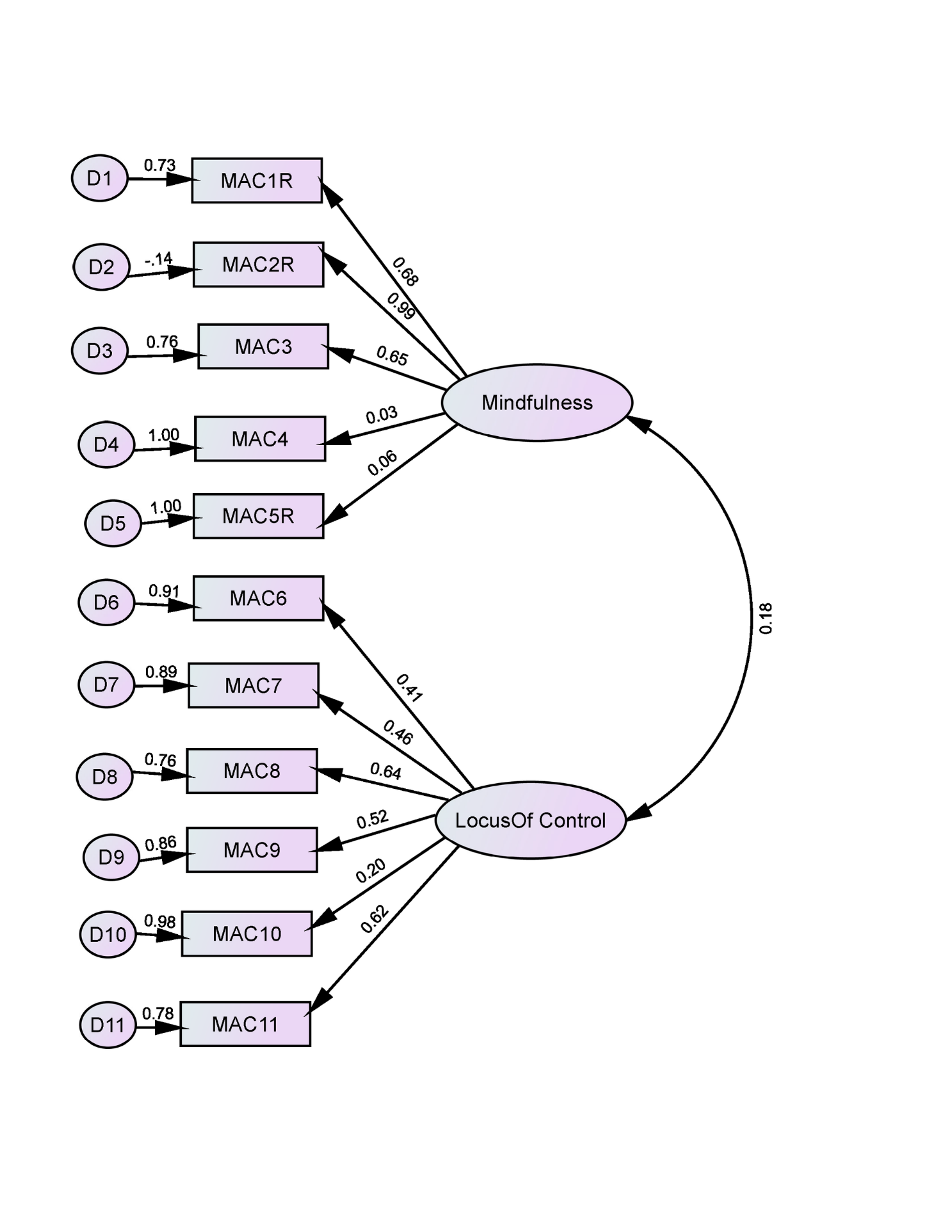


Table S3.4 shows the goodness of fit statistics for both CFA tests. The CFI and NFI indices improved for the second CFA, but only the chi-square/df value indicated an acceptable fit. We can infer that the unacceptable results for Locus of Control detrimentally affected the other goodness of fit indices.

**Table S3.3: CFA statistical results for MAC scale**

|  | **Original CFA** | **Second CFA** | **Internal Consistency**  **Reliability** | **Average**  **Variance**  **Explained** |
| --- | --- | --- | --- | --- |
| **Thought Processing** |  |  | 0.819 | 0.625 |
| MAC1R | 0.68 | 0.73 |  |  |
| MAC2R | 0.99 | 0.93 |  |  |
| MAC3 | 0.65 | 0.69 |  |  |
| **Locus of Control** |  |  | 0.593 | 0.373 |
| MAC8 | 0.65 | 0.60 |  |  |
| MAC9 | 0.52 | 0.50 |  |  |
| MAC11 | 0.63 | 0.71 |  |  |

**Table S3.4: Goodness-of-fit indices for MAC scale**

| **Fit Index** | **Original CFA** | **Second CFA** |
| --- | --- | --- |
| Chi-square | 72.412 | 13.96 |
| df | 43 | 8 |
| p | .003 | .083 |
| Chi-square/df | 1.68 | 1.75 |
| CFI | 0.529 | 0.840 |
| NFI | 0.384 | 0.732 |
| RMSEA | 0.165 | 0.173 |

The Risk scale had no subscales (Figure S3.3). The results of the CFA showed that all seven items did not load well on the construct. Two items (Risk4R and Risk5) loaded negatively, and Risk1R had a relatively low value. These three items were dropped in a subsequent CFA. Loadings increased slightly for Risk2R and Risk3R; they decreased slightly for Risk6 and Risk7. The resulting internal consistency reliability score was 0.869, and the AVE value was 0.649, both at acceptable levels. Table S3.5 compares the goodness of fit statistics for the CFA tests. For the second CFA, the values chi-square/df, CFI, and NFI indicated an acceptable fit, but the RMSEA values were too large.

**Table S3.5: Goodness-of-fit indices for Risk Propensity scale**

| **Fit Index** | **Original CFA** | **Second CFA** |
| --- | --- | --- |
| Chi-square | 23.392 | 3.231 |
| df | 14 | 2 |
| p | .054 | .199 |
| Chi-square/df | 1.67 | 1.62 |
| CFI | 0.908 | 0.945 |
| NFI | 0.810 | 0.945 |
| RMSEA | 0.164 | 0.157 |

**Figure S3.3: Result of Risk Propensity scale CFA test.**


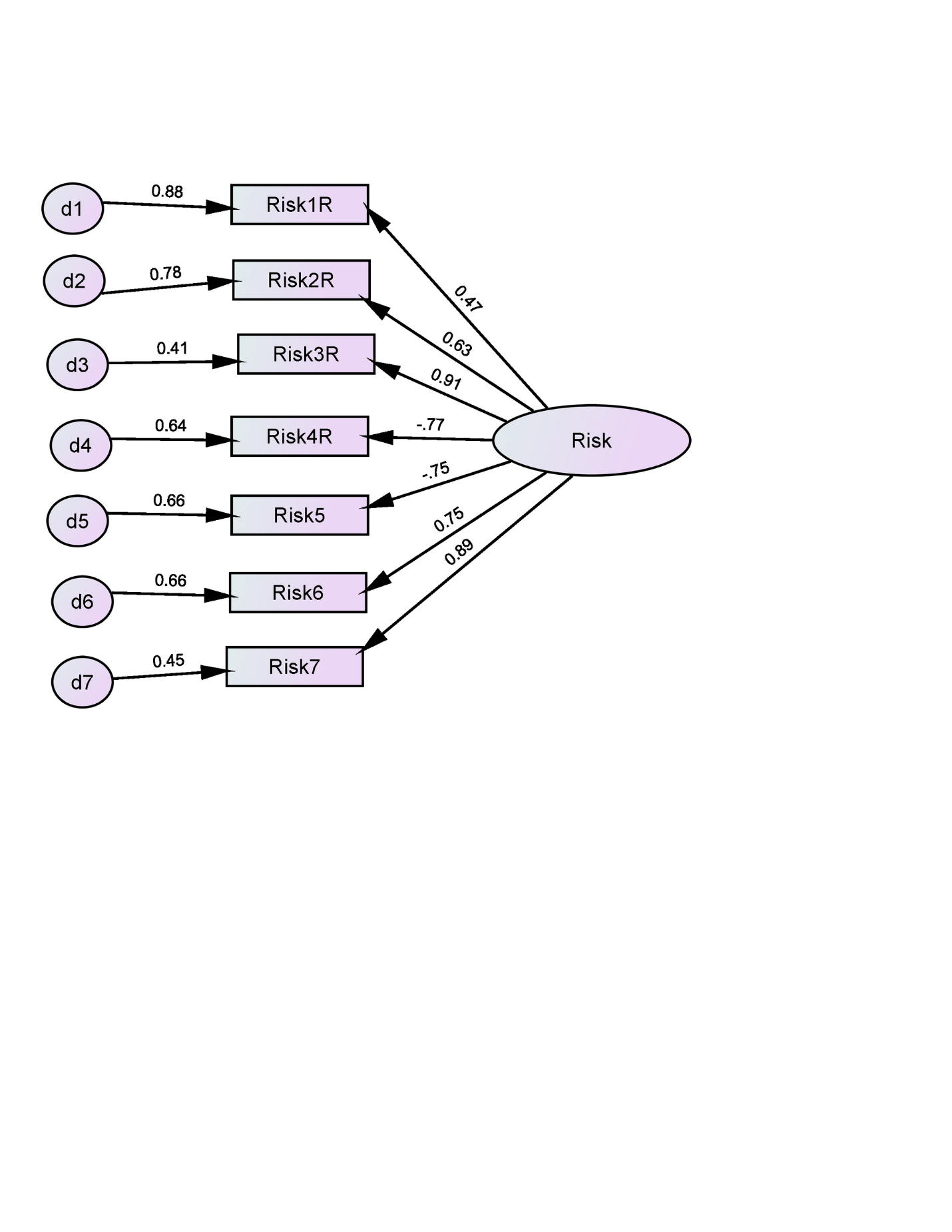

Supplement: S3 Appendix — (DOCX) [file pone.0300497.s003.docx]
