# Supplementary material for: Discovering why people believe disinformation about healthcare
Source: PLoS One. 2024 Mar 21;19(3):e0300497. doi: 10.1371/journal.pone.0300497 (PMC10956743; doi:10.1371/journal.pone.0300497)
Supplement: S4 Appendix — (DOCX) [file pone.0300497.s004.docx]

**Codes Derived from Responses to Open-Ended Questions about Rationale**

| **Raw code** |  | **Condensed list** | |  |
| --- | --- | --- | --- | --- |
| antibiotics for bacteria |  | **Reliable** | | 1 |
| appeal |  | 1 | reliable ad |  |
| based on experience |  | 2 | reliable article |  |
| both dishonest |  | 3 | reliable claims |  |
| both honest |  | 4 | news article |  |
| cannot decide |  |  |  |  |
| CDC |  | **Source** | | 2 |
| cited |  | 5 | reliable/credible source |  |
| claims of clinical tests |  | 6 | CDC |  |
| credibility |  | 7 | FDA |  |
| deeper explanation |  | 8 | Harvard |  |
| detail |  | 9 | doctor |  |
| doctor |  |  |  |  |
| does not include extra words |  | **Ambiguity** | | 3 |
| doesn't cite bible |  | 10 | both dishonest |  |
| false post based on opinion |  | 11 | both honest |  |
| false post from random person |  | 12 | cannot decide |  |
| false post is biased ad |  | 13 | no response |  |
| false post is cocktail |  |  |  |  |
| false post is obviously dishonest |  | **External evidence** | | 4 |
| false post looked like a joke |  | 14 | cited |  |
| false post looks fake |  | 15 | familiarity |  |
| false post not accurate |  | 16 | links |  |
| false post too good to be true |  | 17 | verified |  |
| false post unclear |  | 18 | shared |  |
| false post unprofessional |  | 19 | rights reserved indication |  |
| false post uses buzzwords to sell |  |  |  |  |
| familiarity |  | **20 Photo** | | 20 |
| FDA |  |  |  |  |
| good advice |  | **21 Credibility** | | 21 |
| good grammar |  |  |  |  |
| Harvard |  | **Science** | | 5 |
| included side effects |  | 22 | antibiotics for bacteria |  |
| invalid date |  | 23 | claims of clinical tests |  |
| links |  | 24 | included side effects |  |
| looks possible |  | 25 | not anti-vax |  |
| more appealing |  | 26 | claims can be proven |  |
| more honest than false post |  |  |  |  |
| more practical |  | **Look and feel of honest post** | | 6 |
| more text |  | 27 | appeal |  |
| news article |  | 28 | professional look |  |
| no celebrity |  | 29 | looks possible/promising |  |
| no promises |  | 30 | more appealing |  |
| no response |  | 31 | humility |  |
| not anti-vax |  | 32 | handwriting |  |
| not random |  |  |  |  |
| personal story is less credible |  | **Look and feel of false post** | | 7 |
| photo |  | 33 | false post is biased ad |  |
| professional look |  | 34 | false post is cocktail |  |
| reliable ad |  | 35 | false post is obviously dishonest |  |
| reliable article |  | 36 | false post looked like a joke |  |
| reliable claims |  | 37 | false post looks fake |  |
| reliable source |  | 38 | false post too good to be true |  |
| rights reserved indication |  | 39 | false post unclear |  |
| shared |  |  |  |  |
| unsubstantiated claims |  | **Detail** | | 8 |
| verified |  | 40 | deeper explanation |  |
| false post has no credentials |  | 41 | detail |  |
| claims can be proven |  | 42 | does not include extra words |  |
| humility |  | 43 | more text |  |
| handwriting |  | 44 | based on experience |  |
| targets specific people |  |  |  |  |
|  |  | **Indicators of honest content** | | 9 |
|  |  | 45 | doesn't cite bible |  |
|  |  | 46 | good advice |  |
|  |  | 47 | good grammar |  |
|  |  | 48 | more honest than false post |  |
|  |  | 49 | more practical |  |
|  |  | 50 | no celebrity |  |
|  |  | 51 | no promises |  |
|  |  | 52 | not random |  |
|  |  | 53 | targets specific people |  |
|  |  |  |  |  |
|  |  | **Indicators of dishonest content** | | 10 |
|  |  | 54 | unsubstantiated claims |  |
|  |  | 55 | invalid date |  |
|  |  | 57 | personal story is less credible |  |
|  |  | 58 | false post based on opinion |  |
|  |  | 59 | false post from random person |  |
|  |  | 60 | false post unprofessional |  |
|  |  | 61 | false post uses buzzwords to sell |  |
|  |  | 62 | false post not accurate |  |
|  |  | 63 | false post has no credentials |  |
