# Supplementary material for: Discovering why people believe disinformation about healthcare
Source: PLoS One. 2024 Mar 21;19(3):e0300497. doi: 10.1371/journal.pone.0300497 (PMC10956743; doi:10.1371/journal.pone.0300497)
Supplement: S5 Appendix — (DOCX) [file pone.0300497.s005.docx]

**Additional Data Analysis Results:**

**AOIs as Independent Variables and ‘Correct’ as Dependent Variable**

**1) Key Words Only (19 social media posts)**

**Type III Tests of Fixed Effects (DV: Correct)**

| Source | Numerator df | Denominator df | F | Sig. |
| --- | --- | --- | --- | --- |
| Intercept | 1 | 276.805 | 608.289 | .000 |
| KTVSQRT | 1 | 66.004 | 19.253 | .000 |
| KFixSQRT | 1 | 41.795 | 19.432 | .001 |
| KRevisitsSQRT | 1 | 285.500 | .121 | .729 |

**Estimates of Fixed Effects (DV: Correct)**

|  | | | | | | 95% Confidence Interval | |
| --- | --- | --- | --- | --- | --- | --- | --- |
| Parameter | Estimate | Std. Error | df | t | Sig. | Lower Bound | Upper Bound |
| Intercept | 1.319619 | .053505 | 276.805 | 24.664 | .000 | 1.214291 | 1.424947 |
| KTVSQRT | .194837 | .044404 | 65.181 | 4.388 | .000 | .106181 | .283492 |
| KFixSQRT | -.110888 | .025155 | 41.357 | -4.408 | .000 | -.161660 | -.060116 |
| KRevisitsSQRT | -.008295 | .023890 | 291.550 | -.347 | .729 | -.055319 | .038728 |

**2) Key Words and Sources (17 social media posts)**

**Type III Tests of Fixed Effects (DV: Correct)**

| Source | Numerator df | Denominator df | F | Sig. |
| --- | --- | --- | --- | --- |
| Intercept | 1 | 217.121 | 304.830 | .000 |
| KTVSQRT | 1 | 106.864 | 24.737 | .000 |
| KFixSQRT | 1 | 132.822 | 22.931 | .000 |
| KRevisitsSQRT | 1 | 290.091 | .728 | .394 |
| STVSQRT | 1 | 201.000 | 3.220 | .074 |
| SFixSQRT | 1 | 232.729 | 5.276 | .023 |
| SRevisitsSQRT | 1 | 290.451 | 6.056 | .014 |

**Estimates of Fixed Effects (DV: Correct)**

|  | | | | | | 95% Confidence Interval | |
| --- | --- | --- | --- | --- | --- | --- | --- |
| Parameter | Estimate | Std. Error | df | t | Sig. | Lower Bound | Upper Bound |
| Intercept | 1.316340 | .075394 | 217.121 | 17.459 | .000 | 1.167741 | 1.464939 |
| KTVSQRT | .260655 | .052407 | 106.864 | 4.974 | .000 | .156762 | .364548 |
| KFixSQRT | -.163239 | .034089 | 132.822 | -4.789 | .000 | -.230667 | -.095811 |
| KRevisitsSQRT | .025914 | .030378 | 290.091 | .853 | .394 | -.033875 | .085702 |
| STVSQRT | -.142441 | .079374 | 201.000 | -1.795 | .074 | -.298953 | .014071 |
| SFixSQRT | .133743 | .058227 | 232.729 | 2.297 | .023 | .019024 | .248461 |
| SRevisitsSQRT | -.088043 | .035777 | 290.451 | -2.461 | .014 | -.158459 | -.017628 |

**3) Key Words, Sources, and Photos (13 social media posts)**

**Type III Tests of Fixed Effects (DV: Correct)**

| Source | Numerator df | Denominator df | F | Sig. |
| --- | --- | --- | --- | --- |
| Intercept | 1 | 191.277 | 182.929 | .000 |
| KTVSQRT | 1 | 83.515 | 15.068 | .000 |
| KFixSQRT | 1 | 101.895 | 12.834 | .001 |
| KRevisitsSQRT | 1 | 211.659 | .482 | .488 |
| STVSQRT | 1 | 182.652 | 3.149 | .078 |
| SFixSQRT | 1 | 188.456 | 4.694 | .032 |
| SRevisitsSQRT | 1 | 188.597 | 4.703 | .031 |
| PTVSQRT | 1 | 55.583 | .130 | .719 |
| PFixSQRT | 1 | 84.178 | .153 | .697 |
| PRevisitsSQRT | 1 | 79.414 | .337 | .563 |

**Estimates of Fixed Effects (DV: Correct)**

|  | | | | | | 95% Confidence Interval | |
| --- | --- | --- | --- | --- | --- | --- | --- |
| Parameter | Estimate | Std. Error | df | t | Sig. | Lower Bound | Upper Bound |
| Intercept | 1.385399 | .102432 | 191.277 | 13.525 | .000 | 1.183359 | 1.587440 |
| KTVSQRT | .257401 | .066310 | 83.515 | 3.882 | .000 | .125524 | .389277 |
| KFixSQRT | -.162393 | .045330 | 101.895 | -3.582 | .001 | -.252307 | -.072480 |
| KRevisitsSQRT | .026543 | .038233 | 211.650 | .694 | .488 | -.048823 | .101909 |
| STVSQRT | -.162904 | .091230 | 182.652 | -1.775 | .078 | -.341905 | .018096 |
| SFixSQRT | .150953 | .069673 | 188.456 | 2.167 | .032 | .013514 | .288392 |
| SRevisitsSQRT | -.093758 | .043234 | 188.597 | -.2.169 | .031 | -.179043 | -.008473 |
| PTVSQRT | .023929 | .066251 | 55.583 | .361 | .719 | -.108811 | .156668 |
| PFixSQRT | -.027678 | .045262 | 84.178 | -.391 | .697 | -.107683 | .072326 |
| PRevisitsSQRT | -.17465 | .030088 | 79.414 | -.580 | .563 | -.077348 | -.042418 |

**4) Legend for Areas of Interest (AOIs)**

KTVSQRT: Key words total view time (square root transformation)

KFixSQRT: Key words total number of fixations (square root transformation)

KRevisitsSQRT: Key words total number of revisits (square root transformation)

STVSQRT: Source total view time (square root transformation)

SFixSQRT: Source total number of fixations (square root transformation)

SRevisitsSQRT: Source total number of revisits (square root transformation)

PTVSQRT: Photo total view time (square root transformation)

PFixSQRT: Photo total number of fixations (square root transformation)

PRevisitsSQRT: Photo total number of revisits (square root transformation)
